# Science over language: a plea to consider language bias in scientific publishing

**DOI:** 10.62675/2965-2774.20240084-en

**Published:** 2024-07-01

**Authors:** Sebastián González-Dambrauskas, Jorge Ibrain Figueira Salluh, Flávia Ribeiro Machado, Alexandre Tellechea Rotta

**Affiliations:** 1 Red Colaborativa Pediátrica de Latinoamérica Montevideo Uruguay Red Colaborativa Pediátrica de Latinoamérica (LARed Network) - Montevideo, Uruguay.; 2 Facultad de Medicina Universidad de la República Montevideo Uruguay Departamento de Pediatría y Unidad de Cuidados Intensivos de Niños, Centro Hospitalario Pereira Rossell, Facultad de Medicina, Universidad de la República - Montevideo, Uruguay.; 3 Instituto D’Or de Pesquisa e Ensino Rio de Janeiro RJ Brazil Instituto D’Or de Pesquisa e Ensino - Rio de Janeiro (RJ), Brazil.; 4 Brazilian Research in Intensive Care Network São Paulo SP Brazil Brazilian Research in Intensive Care Network (BRICNET) - São Paulo (SP), Brazil.; 5 Hospital São Paulo Escola Paulista de Medicina Universidade Federal de São Paulo São Paulo SP Brazil Intensive Care Department, Hospital São Paulo, Escola Paulista de Medicina, Universidade Federal de São Paulo - São Paulo (SP), Brazil.; 6 Division of Pediatric Critical Care Medicine Duke University Medical Center Durham North Carolina United States Division of Pediatric Critical Care Medicine, Duke University Medical Center -Durham, North Carolina, United States.

*“Under the present system, if Romeo and Juliet had been written by González instead of Shakespeare, this great work would have been rejected.”*

Antonio Herrera, Nature, 1999^(1)^

## Language hegemony in science

Consider the following fictional predicament: you have just finished writing the main manuscript for a study that, from conception to final analysis, took you more than four years to complete. You must now try to get it published in a respected scientific journal to disseminate your findings to a broad audience and advance your academic career. The catch is that all high-impact journals in this fictional world require manuscripts to be written in Mandarin Chinese. From the first draft to the last response to reviewers, all communication must occur in a language in which you are not fluent. The fact that you are fluent in two languages other than your native tongue is of no help because Mandarin is simply not one of them. *Tampoco sabes español como el primer autor de este manuscrito, ou mesmo português como os outros três*. How does this make you feel? That is probably the same sentiment experienced by most researchers on the planet who are not native English speakers every time they submit a manuscript for peer review within a publishing system rooted in the Anglo-Saxon linguistic tradition. This issue is not confined to journals housed in English-speaking countries. For instance, *Critical Care Science*, the official journal of the *Associação de Medicina Intensiva Brasileira* (AMIB) and the *Sociedade Portuguesa de Cuidados Intensivos* (SPCI), requires manuscripts to be submitted either in English or Portuguese (it publishes accepted articles in both languages) but receives a significant number of submissions from countries where neither of these are a native language.^(2,3)^

It is estimated that more than 7 billion people on planet Earth keep alive more than 7,000 languages, of which 23 have at least 50 million speakers.^(4)^ English is only one of them and is spoken as a native language by fewer people than Mandarin Chinese or Spanish. While the majority of the world’s population is monolingual, English language dominance in science and academia over the last century is evident.^(5)^ It follows that most scientists worldwide are not native English speakers, yet they must publish in English should they aspire to showcase their work in a high-impact journal. Over 85% of the global population resides in low- and middle-income countries where English proficiency is less common than in high-income countries. Such disparities may exacerbate the discrepancies in both producing and accessing scientific literature. This issue is particularly pertinent in critical care. For example, among the top four countries with the highest numbers of intensive care unit beds worldwide (United States, Brazil, China, and Germany), only one has English as its native language. This reality has consequential effects.

## Consequences of language bias

There is evidence to suggest that when a manuscript does not meet a journal’s language standards as determined by its gatekeepers (i.e., reviewers, editors) – meaning that it does not reach a “native fluency” level of English – studies are often deemed as having lower scientific quality.^(6)^ This is an example of language bias, and this bias imposes an additional hurdle for scientists whose first language is not English when they try to publish in relevant journals. Language bias exposes these scientists to the risk of manuscript rejection not based on their scientific merit but on the quality of the language in which they were written.

Language bias may manifest as a subtle form of academic segregation, but it comes with substantial costs. For example, researchers who are nonnative English speakers spend considerably greater effort reading, writing, and speaking in a language that is not their own, especially in the early stages of their careers.^(7)^ Scientists from nations with low English proficiency take nearly twice as long to read an article in English compared to their native English-speaking counterparts. They also take 51% more time to write an article, are 12.5 times more likely to be asked to improve their English writing during revision, and face a 2.6 times greater frequency of language-related paper rejections than native English speakers.^(7)^ Essentially, journals published in English are less likely to accept papers by scientists in countries where English is not the primary language.^(8,9)^ Therefore, it is also unsurprising that nearly 30% of early career nonnative English speaker scientists report having elected not to attend an English-language academic conference, and nearly half of them often or always avoid the opportunity to give oral presentations due to language barriers.^(7)^

Linguistic hegemony and language bias – issues that are often overlooked but are undeniably present – can pose significant threats to the scientific community, as language morphs from being a tool of communication and exchange to becoming an obstacle. Consequently, the broader scientific community misses out on knowledge generated by non-English-speaking researchers, and these researchers, in turn, face the injustice of unequal access to the broader audience that only high-impact journals can reach. The result is a potential decrease in global scientific diversity due to a bias favoring English content generated by native English speakers. This bias toward knowledge generated in the wealthiest regions can, in fact, compromise care. Given that the majority of the global population resides in resource-poor settings, caution is needed when extrapolating from data derived in high-resource settings. Providing care for critically ill patients presents unique and additional challenges, calling for distinct strategies for disease prevention, diagnosis, and treatment. As a result, the expertise and knowledge acquired in these settings must be widely shared with health care professionals working under similar conditions.

## Language bias in biomedical journals

Language is truly an evolutionary tool that defines us as a human species and enables communication. Within the scientific arena, however, our north star should be the language of science itself rather than the language in which it is explained. Recognizing, acknowledging, addressing, and overcoming language bias is of crucial importance and must be a priority for the scientific community to ensure equitable access, diversity, and representation across the global scientific landscape.^(10)^ Partnerships between researchers from high and low-resourced settings should be built on collaboration across all research stages, rather than on the misapprehension that including a native English-speaking author would lend greater credibility to the manuscript and enhance its chances of acceptance.

At biomedical journals, it is customary for the Editor-in-Chief to rely heavily on peer reviewers and senior associate editors to provide content expertise. Manuscripts that pass the initial editorial screening are sent to expert reviewers with the expectation that they will be refereed based on scientific merit. Then, the manuscripts that achieve sufficient priority for acceptance for publication undergo a meticulous review by an editor (or dedicated copy editor) for clarity, syntax, grammar, and conformity to the journal’s style. This process should be applied uniformly, regardless of the English language fluency or country of origin of the authors, thus ensuring that the edited prose is commensurate with the standards and expectations of the journal. Ideally, language (bias) should not be a determinant of a manuscript’s acceptance or rejection. However, while not overt, it would be disingenuous to believe that a manuscript with distracting language and poor grammar would be received by reviewers in the same way as an expertly written one. Similarly, it is unrealistic to assume that the negative impression left by the low quality of prose on a reviewer would not implicitly influence their recommendations.

## A call to publisher, editors, and reviewers: stick to the science!

Just as the shift toward open-access publications, and more recently, the movement to reduce or eliminate publication fees, is crucial for democratizing access to reading and publishing science across all fields, including critical care, addressing language bias is equally imperative.^(11)^We urge publishers, editors, and reviewers to conscientiously refrain from allowing subpar syntax and grammar to diminish the quality of the underlying science. We ask for your linguistic tolerance toward manuscripts that have a robust scientific foundation but are hindered by issues in language usage, which, though distracting, can be addressed during the editorial process. To advance scientific knowledge, it is imperative that sound ideas, discoveries, and innovations have the opportunity to be disseminated and heard, regardless of the native language of their originators. We call upon referees and gatekeepers of scientific knowledge to consider this plea in their publication policies and when assessing the merit of manuscripts for publication. We invite all of you to join us in embracing linguistic diversity in science.
