# Supplementary material for: Science over language: a plea to consider language bias in scientific publishing
Source: Crit Care Sci. 2024 Jul 1;36:e20240084en. doi: 10.62675/2965-2774.20240084-en (PMC11239201; doi:10.62675/2965-2774.20240084-en)
Supplement: Translation

# Translation: A ciência acima do idioma: um apelo à consideração do preconceito linguístico na publicação científica

**DOI:** 10.62675/2965-2774.20240084-pt

**Authors:** Sebastián González-Dambrauskas, Jorge Ibrain Figueira Salluh, Flávia Ribeiro Machado, Alexandre Tellechea Rotta

**Affiliations:** 1 Uruguai Red Colaborativa Pediátrica de Latinoamérica (LARed Network) - Montevidéu, Uruguai.; 2 Uruguai Departamento de Pediatria e Unidade de Terapia Intensiva Infantil, Centro Hospitalario Pereira Rossell, Facultad de Medicina, Universidad de la República - Montevidéu, Uruguai.; 3 Brasil Instituto D’Or de Pesquisa e Ensino - Rio de Janeiro (RJ), Brasil.; 4 Brasil Brazilian Research in Intensive Care Network (BRICNET) - São Paulo (SP), Brasil.; 5 Brasil Departamento de Terapia Intensiva, Hospital São Paulo, Escola Paulista de Medicina, Universidade Federal de São Paulo - São Paulo (SP), Brasil.; 6 Estados Unidos Divisão de Medicina Intensiva Pediátrica, Duke University Medical Center -Durham, Carolina do Norte, Estados Unidos.

*“Sob o sistema atual, se Romeu e Julieta tivesse sido escrito por González em vez de Shakespeare, essa grande obra teria sido rejeitada”.*

Antonio Herrera, Nature, 1999^(1)^

## A hegemonia do idioma na ciência

Considere a seguinte situação fictícia: você acaba de escrever o manuscrito principal de um estudo que, desde a concepção até a análise final, levou mais de 4 anos para ser concluído. Agora, você precisa tentar publicá-lo em uma revista científica respeitada para divulgar suas descobertas para um público amplo e avançar em sua carreira acadêmica. Entretanto, os manuscritos para todas as revistas de alto impacto nesse mundo fictício devem ser escritos em chinês mandarim. Desde o primeiro rascunho até a última resposta aos revisores, toda a comunicação deve ocorrer em um idioma no qual você não é fluente. O fato de você ser fluente em dois idiomas além da sua língua nativa não ajuda em nada, pois o mandarim simplesmente não é um deles. Você não sabe espanhol como o primeiro autor deste manuscrito, ou mesmo português como os outros três. Como isso faz você se sentir? Provavelmente, esse é o mesmo sentimento experimentado pela maioria dos pesquisadores do planeta que não são falantes nativos de inglês toda vez que enviam um manuscrito para revisão por pares em um sistema de publicação enraizado na tradição linguística anglo-saxônica. Esse problema não se limita a periódicos sediados em países de língua inglesa. Por exemplo, a *Critical Care Science*, a revista oficial da Associação de Medicina Intensiva Brasileira (AMIB) e da Sociedade Portuguesa de Cuidados Intensivos (SPCI), exige que os manuscritos sejam enviados em inglês ou português (ela publica os artigos aceitos em ambos os idiomas), mas recebe um número significativo de envios de países em que nenhum desses idiomas é nativo.^(2,3)^

Estima-se que mais de 7 bilhões de pessoas no planeta Terra mantenham vivos mais de 7.000 idiomas, dos quais 23 têm pelo menos 50 milhões de falantes.^(4)^ O inglês é apenas um deles e é falado como idioma nativo por menos pessoas do que o chinês mandarim ou o espanhol. Embora a maioria da população mundial seja monolíngue, o domínio da língua inglesa na ciência e no meio acadêmico no último século é evidente.^(5)^ Assim, ainda que a maioria dos cientistas do mundo todo não seja falante nativo de inglês, eles precisam publicar em inglês se quiserem apresentar seu trabalho em uma revista de alto impacto. Mais de 85% da população global reside em países de baixa e média renda, onde a proficiência em inglês é menos comum do que em países de alta renda. Essas disparidades podem exacerbar as discrepâncias tanto na produção quanto no acesso à literatura científica. Essa questão é particularmente pertinente em cuidados intensivos. Por exemplo, entre os quatro principais países com o maior número de leitos de unidades de terapia intensiva do mundo (Estados Unidos, Brasil, China e Alemanha), apenas um tem o inglês como idioma nativo. Essa realidade tem efeitos significativos.

## Consequências do preconceito linguístico

Há evidências sugerindo que, quando um manuscrito não atende aos padrões de idioma de uma revista, conforme determinado por seus guardiões (ou seja, revisores, editores), o que significa que ele não atinge um nível de “fluência nativa” do inglês, os estudos geralmente são considerados de qualidade científica inferior.^(6)^ Esse é um exemplo de preconceito linguístico, e esse preconceito impõe um obstáculo adicional aos cientistas cujo primeiro idioma não é o inglês quando tentam publicar em revistas relevantes. O preconceito linguístico expõe esses cientistas ao risco de seus manuscritos serem rejeitados não com base em seu mérito científico, mas na qualidade do idioma em que foram escritos.

O preconceito linguístico pode se manifestar como uma forma sutil de segregação acadêmica, mas tem custos substanciais. Por exemplo, os pesquisadores que não são falantes nativos de inglês despendem um esforço consideravelmente maior para ler, escrever e falar em um idioma que não é o seu, especialmente nos estágios iniciais de suas carreiras.^(7)^ Cientistas de países com baixa proficiência em inglês levam quase o dobro do tempo para ler um artigo em inglês em comparação com seus colegas falantes nativos de inglês. Eles também levam 51% mais tempo para escrever um artigo, têm 12,5 vezes mais probabilidade de serem solicitados a melhorar sua redação em inglês durante a revisão e enfrentam uma frequência 2,6 vezes maior de rejeições de artigos relacionadas ao idioma do que os falantes nativos de inglês.^(7)^ Essencialmente, as revistas publicadas em inglês têm menos probabilidade de aceitar artigos de cientistas de países onde o inglês não é o idioma principal.^(8,9)^ Portanto, também não é surpreendente que cerca de 30% dos cientistas em início de carreira que não são falantes nativos de inglês afirmem ter optado por não participar de uma conferência acadêmica em inglês, e quase metade deles frequentemente, ou sempre, evita a oportunidade de fazer apresentações orais devido às barreiras do idioma.^(7)^

A hegemonia linguística e o preconceito linguístico – questões muitas vezes ignoradas, mas inegavelmente presentes – podem representar ameaças significativas à comunidade científica, pois o idioma deixa de ser uma ferramenta de comunicação e intercâmbio para se tornar um obstáculo. Consequentemente, a comunidade científica mais ampla carece de conhecimento gerado por pesquisadores que não falam inglês, e esses pesquisadores, por sua vez, enfrentam a injustiça do acesso desigual ao público mais amplo que somente as revistas de alto impacto podem alcançar. Como resultado, a diversidade científica global pode diminuir devido a um viés que favorece o conteúdo em inglês gerado por falantes nativos da língua inglesa. Essa tendência ao conhecimento gerado nas regiões mais ricas pode, de fato, comprometer o atendimento. Considerando que a maioria da população global reside em locais com poucos recursos, é necessário ter cautela ao extrapolar dados derivados de locais com muitos recursos. A prestação de assistência a pacientes em estado crítico apresenta desafios únicos e novos, exigindo estratégias distintas de prevenção, diagnóstico e tratamento de doenças. Como resultado, a experiência e o conhecimento adquiridos nesses ambientes devem ser amplamente compartilhados com os profissionais de saúde que trabalham em condições semelhantes.

## Preconceito linguístico em revistas biomédicas

A língua é realmente uma ferramenta evolutiva que nos define como espécie humana e permite a comunicação. Na arena científica, entretanto, nossa estrela do norte deve ser a linguagem da própria ciência, não a língua em que ela é explicada. Reconhecer, saber, abordar e superar o preconceito linguístico são de importância crucial e devem ser uma prioridade para a comunidade científica para garantir o acesso equitativo, a diversidade e a representação em todo o cenário científico global.^(10)^ As parcerias entre pesquisadores de ambientes com muitos e poucos recursos devem ser construídas com base na colaboração em todos os estágios da pesquisa, em vez de se basearem na percepção errônea de que a inclusão de um autor nativo de língua inglesa daria maior credibilidade ao manuscrito e aumentaria suas chances de aceitação.

Nas revistas biomédicas, é comum que o editor-chefe conte com a ajuda de revisores e editores associados seniores para fornecer conhecimento especializado sobre o conteúdo. Os manuscritos que passam pela triagem editorial inicial são enviados a revisores especialistas com a expectativa de que sejam avaliados quanto ao mérito científico. Em seguida, os manuscritos que obtiverem prioridade suficiente para serem aceitos para publicação passam por uma revisão meticulosa por um editor (ou editor de texto dedicado) quanto à clareza, sintaxe, gramática e conformidade com o estilo da revista. Esse processo deve ser aplicado de maneira uniforme, independentemente da fluência no idioma inglês ou do país de origem dos autores, garantindo que a prosa editada esteja de acordo com os padrões e as expectativas da revista. Idealmente, o idioma (viés) não deve ser fator determinante para a aceitação ou recusa de um manuscrito. No entanto, embora não seja evidente, seria ilusório acreditar que um manuscrito com linguagem confusa e gramática incorreta seria recebido pelos revisores da mesma forma que um manuscrito escrito por um especialista. Da mesma forma, não é realista supor que a impressão negativa deixada pela baixa qualidade do texto em um revisor não influenciaria implicitamente suas recomendações.

## Um apelo a editoras, editores e revisores: atenham-se à ciência!

Assim como a mudança voltada a publicações de livre acesso e, mais recentemente, o movimento para reduzir ou eliminar as taxas de publicação são cruciais para democratizar o acesso à leitura e à publicação de ciência em todos os campos, inclusive no de cuidados intensivos, abordar o preconceito linguístico é igualmente imperativo.^(11)^ Apelamos a editores, editoras e revisores para que se abstenham conscientemente de permitir que a sintaxe e a gramática abaixo da média diminuam a qualidade da ciência subjacente. Pedimos sua tolerância linguística em relação a manuscritos que tenham uma base científica sólida, mas que sejam prejudicados por problemas no uso da língua, que, embora causem distração, podem ser resolvidos durante o processo editorial. Para o avanço do conhecimento científico, é imperativo que ideias, descobertas e inovações sólidas possam ser divulgadas e ouvidas, independentemente do idioma nativo de seus autores. Pedimos aos árbitros e aos guardiões do conhecimento científico que considerem esse apelo em suas normas de publicação e em suas avaliações do mérito dos manuscritos para publicação. Convidamos todos vocês a se juntarem a nós na adoção da diversidade linguística na ciência.
